# The influence of acute stress on attention mechanisms and its electrophysiological correlates

**DOI:** 10.3389/fnbeh.2014.00353

**Published:** 2014-10-09

**Authors:** Jessica Sänger, Laura Bechtold, Daniela Schoofs, Meinolf Blaszkewicz, Edmund Wascher

**Affiliations:** ^1^Experimental Biological Psychology, Institute of Experimental Psychology, Heinrich-Heine-UniversityDüsseldorf, Germany; ^2^Biological Psychology, Institute of Experimental Psychology, Heinrich-Heine-UniversityDüsseldorf, Germany; ^3^Clinic for Psychiatry, Social Psychiatry and Psychotherapy, Hannover Medical SchoolHannover, Germany; ^4^Analytical Chemistry, Leibniz Research Centre for Working Environment and Human FactorsDortmund, Germany; ^5^Perceptual Cybernetics, Leibniz Research Centre for Working Environment and Human FactorsDortmund, Germany

**Keywords:** attention, stress, EEG/ERP, competition, N1/N2pc

## Abstract

For the selection of relevant information out of a continuous stream of information, which is a common definition of attention, two core mechanisms are assumed: a competition-based comparison of the neuronal activity in sensory areas and the top-down modulation of this competition by frontal executive control functions. Those control functions are thought to bias the processing of information toward the intended goals. Acute stress is thought to impair these frontal functions through the release of cortisol. In the present study, subjects had to detect a luminance change of a stimulus and ignore more salient but task irrelevant orientation changes. Before the execution of this task, subjects underwent a socially evaluated cold pressor test (SECPT) or a non-stressful control situation. The SECPT revealed reliable stress response with a significant increase of cortisol and alpha-amylase. Stressed subjects showed higher error rates than controls, particularly in conditions which require top-down control processing to bias the less salient target feature against the more salient and spatially separated distracter. By means of the EEG, subjects who got stressed showed a reduced allocation to the relevant luminance change apparent in a modulation of the N1pc. The following N2pc, which reflects a re-allocation of attentional resources, supports the error pattern. There was only an N2pc in conditions, which required to bias the less salient luminance change. Moreover, this N2pc was decreased as a consequence of the induced stress. These results allow the conclusion that acute stress impairs the intention-based attentional allocation and enhances the stimulus-driven selection, leading to a strong distractibility during attentional information selection.

## Introduction

Every day we get besieged with information of our environment and we are not able to process and respond to all information at once. Attention is one of the most important cognitive functions to deal with this problem. By selecting the most conspicuous or relevant information it helps us to achieve our current behavioral goals.

According to the biased competition account of attentional selection (Desimone and Duncan, 1995), selection is thought to rely on two basic mechanisms. First and foremost information is selected by the intrusive salience of an event, object or feature (for example, whether it is brightly colored, loud or moving; bottom-up). Second, this stimulus-driven competition can additionally be biased by top–down factors such as goals, knowledge, and expectations of the observer. This top-down-induced attentional control has been assigned to fronto-parietal cortical structures that impinge on sensory areas (e.g., Chelazzi et al., 1993, 2001; Reynolds et al., 2000; Reynolds and Desimone, 2003; Connor et al., 2004; Beck and Kastner, 2009).

Evidence for this competition model of attentional selection has been shown by several monkey single-cell studies as well as functional imaging studies. Both types of studies show the same neural response pattern: During directed attention, top-down biasing signals, which originate in fronto-parietal areas, modulate the activity in sensory regions (e.g., Chelazzi et al., 1993, 1998; Kastner et al., 1998; Miller and Cohen, 2001; Gazzaley et al., 2007; Hampshire et al., 2007; Woolgar et al., 2011). Thereby, competitive interactions of top-down and bottom-up directed processes that occur in visual cortex at best comprise the enhancement of the relevant information and by the virtue of the inhibitory interactions necessarily result in a suppression of the irrelevant information (Desimone and Duncan, 1995).

In a series of electrophysiological studies Wascher and various co-workers could show that the initial allocation of attention and orienting to the most salient stimulus in the visual field is accompanied by an enhanced asymmetric N1 (N1pc, Wascher and Beste, 2010a,b; Beste et al., 2011; Sänger and Wascher, 2011; Sänger et al., 2012; Schneider et al., 2012b; Wascher et al., 2012). Furthermore, if the initial allocation of attention was misguided by a very salient distracter stimulus (a bar that changed its orientation) this N1pc was followed by an N2pc toward the relevant stimulus (a bar that changed its luminance). This N2pc is thought to reflect the top-down guided attentional selection toward the relevant information (Eimer, 1996; Wascher and Wauschkuhn, 1996) and can be seen as a correlate of post-selective target processing (Hickey et al., 2009). These processes were accompanied by a fronto-central negativity that arose whenever conflicting information were presented and which was even enhanced if participants put more effort on the task (Wascher and Beste, 2010a,b; Sänger and Wascher, 2011).

Beside these findings the P3, a later ERP component that is largely insensitive to sensory modality, has been conceptualized as an index of selective attention that reflects processes involved in evaluating targets to engage appropriate goal-directed responses (Nieuwenhuis et al., 2005; Polich, 2007). Most of the results of ERP and fMRI studies investigating the P3 used various kinds of oddball tasks, which are different to the experimental design of the present study. However, they suggest that the P3a reflects frontal attentional functioning and thereby implying top-down control (e.g., Daffner et al., 2000; Debener et al., 2002, 2005; Bledowski et al., 2004), what can also be interpreted in a wider frame, where focal attention is needed for stimulus evaluation/discrimination. And indeed, the P3 amplitude is attenuated if a secondary task comes into play or regarding distractions (Kok, 2001; for a review see Linden, 2005; Polich, 2007).

Although stress is one of the most important factors affecting our daily life, there are only few studies that have addressed the link between stress and attentional processing with rather inconclusive results. In those studies using electroencephalography (EEG) or magneto encephalography (MEG, Shackman et al., 2011; Elling et al., 2012) stress led to an enhanced N1 and N1m respectively, which was assumed to be an index of increased exogenous attention processing. However, beside some puzzling effects due to the order of the stress and control condition when using a within-subject design, both studies did neither continuously control for the hypothalamus-pituitary-adrenal (HPA) or catecholaminergic responsitivity by means of cortisol or alpha-amylase nor did they report conclusive behavioral performance decreases that would have to go along with an enhanced distractibility and support the electrophysiological results. Thus, although they indisputably show that stress had an impact on early exogenous attention processing by means of EEG and MEG, it still remains unclear how stress alternates attention processing in particular and how this might result in, e.g., altered behavioral performance.

Due to its extensive connections with sensory and motor cortices the dorsolateral prefrontal cortex (dlPFC) is thought to be a key structure for regulating attention, thought and action (Goldman-Rakic, 1987; Robbins, 2000). It has been suggested that in situations of acute stress PFC functioning is affected (Schoofs et al., 2009; for a review, see Arnsten, 2009). There are two possible pathways how stress may detract the PFC-dependent cognitive control. First, via an increased activity of the sympathetic nervous system (SNS) stress leads to a rapid and short lasting increase in catecholamine release. By that, activation of noradrenergic and dopaminergic projections emanating from the locus coeruleus (LC), which is one of the most stress-sensitive structures in the brain (Herman and Cullinan, 1997), and the tegmental field (VTA—ventral tegmental area) result in a decreased firing rate of PFC neurons (Ramos et al., 2006; Ramos and Arnsten, 2007; Vijayraghavan et al., 2007; Arnsten, 2009). Furthermore, the LC plays an essential role in the inhibition of spontaneous orienting responses to distracting stimuli (Aston-Jones and Cohen, 2005; Benarroch, 2009). In case of an increased tonic LC activity, as it becomes evident under acute stress, this may impair the inhibiting phasic input from the LC on other cortical areas that are involved in the orienting to distracting stimuli (Corbetta and Shulman, 2002; Pessoa et al., 2003) and result in an increased distractibility and a deficient focused attention (Valentino and Van Bockstaele, 2008; Benarroch, 2009).

While the first and fast SNS-mediated stress response, which can be measured, e.g., by salivary alpha-amylase (sAA, for a review see Nater and Rohleder, 2009; Rohleder and Nater, 2009), is temporally linked to the subjective stress experience (e.g., feeling of increased arousal and bad mental state) the second pathway via the HPA axis comes into play rather prolonged, when subjective stress levels are already back to baseline levels (Plessow et al., 2011, 2012a,b). This increased activity of the HPA axis leads to the synthesis and increased release of glucocorticoids (mainly cortisol, e.g., de Kloet et al., 2005). Glucocorticoids like cortisol primarily bind to mineralcorticoid and glucocorticoid receptors which are abound in the PFC (e.g., Perlman et al., 2007) and thus lead to alterations in prefrontal brain activity (Arnsten, 2009; Qin et al., 2009; Weerda et al., 2010).

However, studies that report stress-related alterations in behavioral performance report very contradictive results. While some report an improvement of attentional and/or cognitive control functioning in situations of acute stress (e.g., Wells and Matthews, 1994; Chajut and Algom, 2003; Kofman et al., 2006; Weerda et al., 2010; Beste et al., 2013), others show that stress disrupts mechanisms involved in attentional and/or cognitive control functioning (e.g., Steinhauser et al., 2007; Arnsten, 2009; Plessow et al., 2012a).

Although factors like the glucocorticoid sensitivity that varies with gonadal steroids release (Schoofs and Wolf, 2009) might have led to the different effects, the most conspicuous difference might be due to the duration of the time lag between stress induction and experimental testing of the attentional and/or cognitive control function. Supported by the findings of Plessow et al. (2011), who report a stress-induced time-dependent decrease of cognitive flexibility, acute stress effects on cognitive control processes can be closely linked to the HPA-stress response time course. While most of the studies that report performance increases or absent decreases used a short or no time lag to the stressor, longer time lags go along with decreased cognitive control performance.

Thus, depending on the HPA-stress response time course, stress impairs the PFC influence on attention processing. By that, attentional selection switches from a thoughtful top-down control by the PFC that is based on what is most relevant to the task at hand to a bottom-up control by the sensory cortices (Arnsten, 2009), whereby the salience of the stimulus dominates the attentional selection (Buschman and Miller, 2007; Mather and Sutherland, 2011; Sutherland and Mather, 2012).

In the present study, we investigated which processing stages of the attentional competition are impaired by acute stress, induced by the Socially Evaluated Cold Pressor Test (SECPT, Schwabe et al., 2008). As outlined above, it can be assumed that in situations of acute stress the noradrenergic and dopaminergic influence on the PFC leads to a lack of top-down modulation of attentional selection. Thus, the exogenous attention (bottom-up) will dominate the selection processing and will result in deficient controllability of distracter stimuli.

To examine the different stages of attentional selection we employed a change detection task, similar to the one used by Wascher and Beste (2010b). The subject's task was to detect changes of luminance and to ignore changes of orientation during trials in which the stimulus dimensions could either change singularly or simultaneously and spatially separated or joint. If both luminance and orientation change simultaneously but spatially separated, a perceptual conflict is induced in which subjects have to enhance the processing of the less salient but task relevant luminance change against the competing and more salient orientation change.

We propose that under acute stress PFC top-down control will be switched off and attentional selection gets dominated by the bottom-up saliency of the stimuli. Furthermore, the lack of top-down control should be reflected by an alteration of early and late fronto-central EEG activity (N2 and P3a) and an increase in response errors when distracting stimuli are present. The attentional conflict should also go along with an increased N1pc toward distracting stimuli and the absence of the following N2pc as an index of post-selective target processing.

## Materials and methods

### Participants

Twenty-four male volunteers between 19 and 35 years participated in the present study (mean age = 24.86, *SD* = 4.43 years). They were all in good physical health, medication-free, normally weighted (as indicated by the body-mass index, BMI, 17 < BMI < 28; mean BMI = 23.11, *SD* = 2.25), non-smokers, right-handed and with normal or corrected-to-normal vision. Only men were included to avoid possible gender and ovarian cycle effects on adrenocortical reactivity (see Kirschbaum et al., 1999; Kudielka and Kirschbaum, 2005, for a review). None of the participants reported any acute or chronic stress. None of them had a history of neurological or psychiatric disorders. Moreover, participants were asked to refrain from excessive exercise, meals, and caffeine within 2 h before the experimental sessions. The volunteers were recruited by announcements and received financial compensation or course credits, respectively. All of them gave their written informed consent prior to their inclusion in the study. The study was approved by the local ethics committee of the Ruhr-University of Bochum and conducted in accordance to ethical standards of the 1964 Declaration of Helsinki.

### Stress protocol and control condition

In a between-subject design, participants were assigned to either the stress or control condition. Both groups were matched regarding age and BMI. Participants in the stress condition (*n* = 12) were exposed to the Socially Evaluated Cold Pressor Test (SECPT, Schwabe et al., 2008). The SECPT has been used in several studies as an efficient stress induction method that leads to significant elevations in autonomic arousal, salivary cortisol, salivary alpha-amylase (sAA) and subjective stress ratings (Schwabe et al., 2009, 2010; Schwabe and Wolf, 2009, 2010). This test included the immersion of their right or left forearm, excluding the hand, for 3 min (or until they could no longer tolerate it) into ice water (0–3°C). During forearm immersion, they were instructed to look into a camera. Participants were told that these video recordings would later be analyzed for facial expression and will be compared with the recordings of the other participants of the study. Furthermore, participants were monitored by a female experimenter.

In the warm water control condition, participants (*n* = 12) submerged their right or left forearm, excluding the hand, for 3 min in warm water (35–37°C); they were neither video-taped nor monitored by the experimenter.

Both groups were comparable in the time they kept their hands in the water (Control: *M* = 163.05 s, *SD* = 12.05; Stress: *M* = 161.44 s, *SD* = 12.05; *F* < 1). Immediately after the SECPT subjective stress ratings were collected using a visual analog scale (VAS) to validate the efficacy of the SECPT. Therefore, participants rated on an 11-point scale ranging from 0 (“not at all”) to 10 (“very much”) how painful the previous situation had been.

### Stimuli and procedure

**Figure 1:**
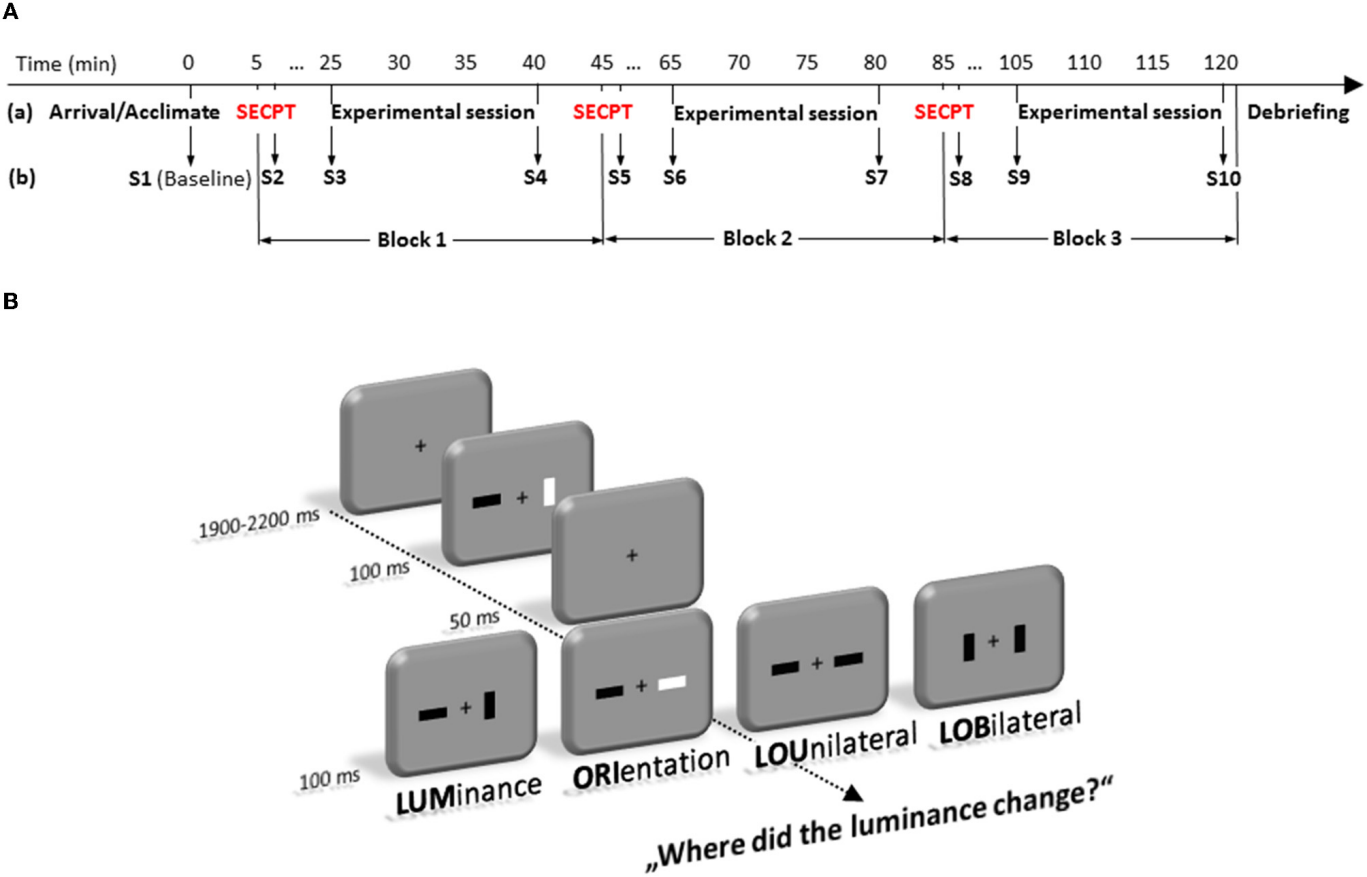
**Time-line (in minute) of the experimental protocol (A)**. It depicts the sequence of stress and experimental test sessions **(a)** as well as the 10 time points where saliva samples (S1–S10) were taken **(b)**. **(B)** represents the experimental paradigm. Participants had to detect changes in luminance of a bar in a fast sequence of frames that could occur alone (LUM), accompanied by a change in orientation of the same bar (LOU), accompanied by an orientation change at the opposite location (LOB). Trials in which only the orientation (ORI) changed at a single bar were no-go trials.

Each trial started with a fixation cross that was presented between 1900 and 2200 ms in the center of the screen. Then two bars, which were either darker or brighter than the background (30 cd/m^2^), and could be oriented horizontally or vertically, were presented left and right from a fixation cross (see Figure 1). Each trial consisted of two frames which were presented in fast succession. Each frame was shown for 100 ms, interrupted by a short blank of 50 ms, in which only the fixation cross was visible. Luminance and orientation of the bars were randomly intermixed in any possible combination for the first frame. From first to second frame, either the luminance (LUM) or the orientation (ORI) of one single bar, luminance and orientation of one bar (LOU = Luminance-Orientation Unilateral), or luminance and orientation distributed across the two bars (LOB = Luminance-Orientation Bilateral) could change. Participants' task was to indicate where the luminance of a bar had changed. Therefore, they had to press one out of two buttons of a custom made response box (RB-620, Cedrus Corporation, San Pedro, USA) with the index finger of the left or right hand at the side where the change had appeared. The LOB condition will also be referred to as the “conflict” condition, because in this condition relevant and irrelevant information are spatially separated.

Trials in which only the orientation of one bar had changed were no-go trials. All participants were instructed to respond as fast as possible but not at the cost of accuracy.

Each experimental session consisted of 384 trials and took approximately 15 min. There was a rest period in between the three experimental blocks. Thus, for each condition 96 trials were presented in random order. Stimulus presentation and behavioral data recording was controlled by Presentation (Version 11; Neurobehavioral Systems Inc., Albany, USA).

During the task, all participants were seated in a comfortable armchair inside a dimly lit, sound attenuated and electrically shielded room in front of a 20″ computer monitor (Mitsubishi—DiamondPro 2070SB), that subtended 14.03 × 18.65° at the viewing distance of 120 cm.

To avoid any influence of the circadian profiles of adrenocortical reactivity and cognitive ability, all measurements were conducted in the afternoon (starting between 14:00 and 15:00 p.m.). After a participant's arrival, pre-experimental saliva (cortisol and alpha-amylase) measurements were taken. Then, participants were exposed either to the SECPT or to the warm water control condition. Immediately thereafter, subjective assessments of the previous situation were measured again. Twenty min after the SECPT/control treatment (before the experimental session started) and after the experimental session, saliva (cortisol and alpha-amylase) measurements were taken again. All subjects performed these SECPT/control procedure three times (= 10 saliva samples from each participant, see Figure 1A).

### Data recording and analysis

#### Cortisol and alpha-amylase data

Saliva samples were obtained by the use of Salivettes (Sarstedt, Nümbrecht, Germany). Salivary free cortisol and sAA levels were obtained using a chemiluminescence immunoassay (IBL International, Hamburg, Germany) as described in detail by Dressendorfer et al. (1992). Samples were taken directly when participants entered the lab (baseline), directly after cessation of each stressor (+3 min, S2, S5, and S8) as well as 20 (+20 min, S3, S6, and S9) and 35 (+35 min, S4, S7, and S10) min later respectively (see Figure 1A). For further statistical analysis, both, salivary free cortisol and sAA are reported as delta increases relative to baseline measurements. Two ANOVAs were run which included the within-subject factors Experimental block (3, block1, block2, block3), Time (3, +3 min, +20 min, +35 min) and the between-subject factor Group (2, control vs. stress).

#### Behavioral data

Response times (RTs) were measured from the onset of the second frame until the button press. Pressing the button too quickly (< 80 ms) was defined as a premature response error. Further substantial response errors could be wrong button presses, false alarms in the no-go condition, and misses (no response within 1500 ms). All error trials were excluded from response time and EEG analyses. Global accuracy was tested in an ANOVA with the repeated-measurement factor Type of change (LUM, ORI, LOU, LOB), and the between-subject factor Group (stress vs. control). RT analysis was restricted to correct responses in those conditions in which subjects had to respond. Thus, this analysis consisted of only three types of change (LUM, LOU, LOB) for the two groups (stress and control).

#### EEG data

The electroencephalogram (EEG) was recorded with a QuickAmp (Brain Products, Herrsching, Germany) DC-amplifier set at 200 Hz low pass filtering from 45 scalp positions according to the extended 10/20 System (Pivik et al., 1993) using an EEG recording cap and Ag/AgCl ring electrodes (EASYCAP, Munich, Germany). Sampling rate was set to 1 kHz. During acquisition, common average served as reference. Data were re-referenced offline to linked mastoids (TP9 and TP10). A ground electrode was affixed at the forehead (AFz). For recognizing ocular artifacts, the vertical and horizontal electrooculogram (EOG) was recorded bipolarly from the outer canthi of the two eyes and above vs. below the right eye respectively.

Segments with a length of 2200 ms (−200–2000 ms with respect to the onset of first stimulus) were defined for further processing. Baseline was set to 200 ms preceding the first frame. These selected segments were checked offline for artifacts (zero-lines, fast shifts, and drifts). Trials with horizontal eye movements (saccades) preceding the latency of the components of interest (up to 400 ms after the change) were excluded by manual inspection. The influences of remaining eye movements (e.g., blinks) on electrocortical activity were corrected by the algorithm proposed by Gratton and co-workers (Gratton et al., 1983).

Further analysis included only trials with correct responses. Due to the fast sequence of the two frames, ERP responses of the two stimuli, in particular sensory components did largely overlap. Thus, to address visuo-spatial processing, posterior (PO7/PO8) event-related asymmetries of the EEG were calculated (event-related lateralization's, ERLs = contralateral minus ipsilateral activity) like a lateralized readiness potential is computed (Coles et al., 1988; Wascher and Wauschkuhn, 1996). These responses should be restricted to the occurrence of the change (second frame) since stimulation is bilateral up to this point. Bottom-up driven activations of the sensory system has been determined in the N1 range. These were measured as the maximum of asymmetry between 150 and 250 ms in the ERL after the second stimulus. As an equivalent of the N2pc, the asymmetry following that first response was measured as the maximum of asymmetry in a time window between 250 and 350 ms. Both components (N1pc and N2pc) were first tested in an *t*-test against zero and afterwards tested in an ANOVA including the within-subject factor Type of change (4, LUM vs. ORI vs. LOU vs. LOB) and the between-subject factor Group (2, stress vs. control).

In the regular ERP, the maximal effect of stress has been observed at fronto-central sites. Thus, statistical analyses of the N2 and P3a included the same factors as for the N1pc and N2pc (Type of change and Group) but were restricted to the fronto-central site FCz. There, an enhanced negative going ERP appeared that was maximal if participants got stressed. Since there were no distinct peaks, N2 and P3a were measured as the mean amplitude between 170 and 220 ms (N2), 250 and 300 ms (P3a).

In case of a violation of the assumption of sphericity for analyses with more than one degree of freedom data were Greenhouse-Geisser corrected (cf. Vasey and Thayer, 1987). In that case, corrected *p*-values, (uncorrected) degrees of freedom, and the Greenhouse–Geisser estimate (ϵ, epsilon) are reported. Planned comparisons were computed as follow-up tests to break down the omnibus ANOVA effects. Any additional *post-hoc* analyses are described in the text below.

In order to test whether changes in cortisol or sAA were directly associated with the behavioral performance (error rates or response times), electrophysiological activity of the PFC (fronto-central N2 and P3) or posterior attention-related components (N1pc and N2pc) correlations were calculated for each Type of change (LUM, ORI, LOU, and LOB) using delta increases for the neuroendocrine measures (post treatment minus baseline). For the cortisol- and sAA-level delta increases were defined as the mean concentration on the +3 min (S2, S5, S8), +20 min (S3, S6, S9) and +35 min (S4, S7, S10) measurement minus baseline. Because one participant of the stress group showed high absolute as well as high relative cortisol levels, we only report and graphically present those correlations, which were still significant when excluding him. Due to the small sample size and the different scales of the measures that were entered into the analysis, Spearman's Rho (ρ) with one-tailed significance level is reported. For the sake of perspicuity, only significant effects are reported.

## Results

### Subjective and physiological responses to stress

Participants' subjective ratings and cortisol changes indicated that stress was successfully induced by the SECPT. By means of the VAS participants of the SECPT group rated the forearm immersion as significantly more painful (5.67) than participants in the warm water control group [0.94; *F*_(1, 22)_ = 61.94, *p* < 0.001].

**Figure 2:**
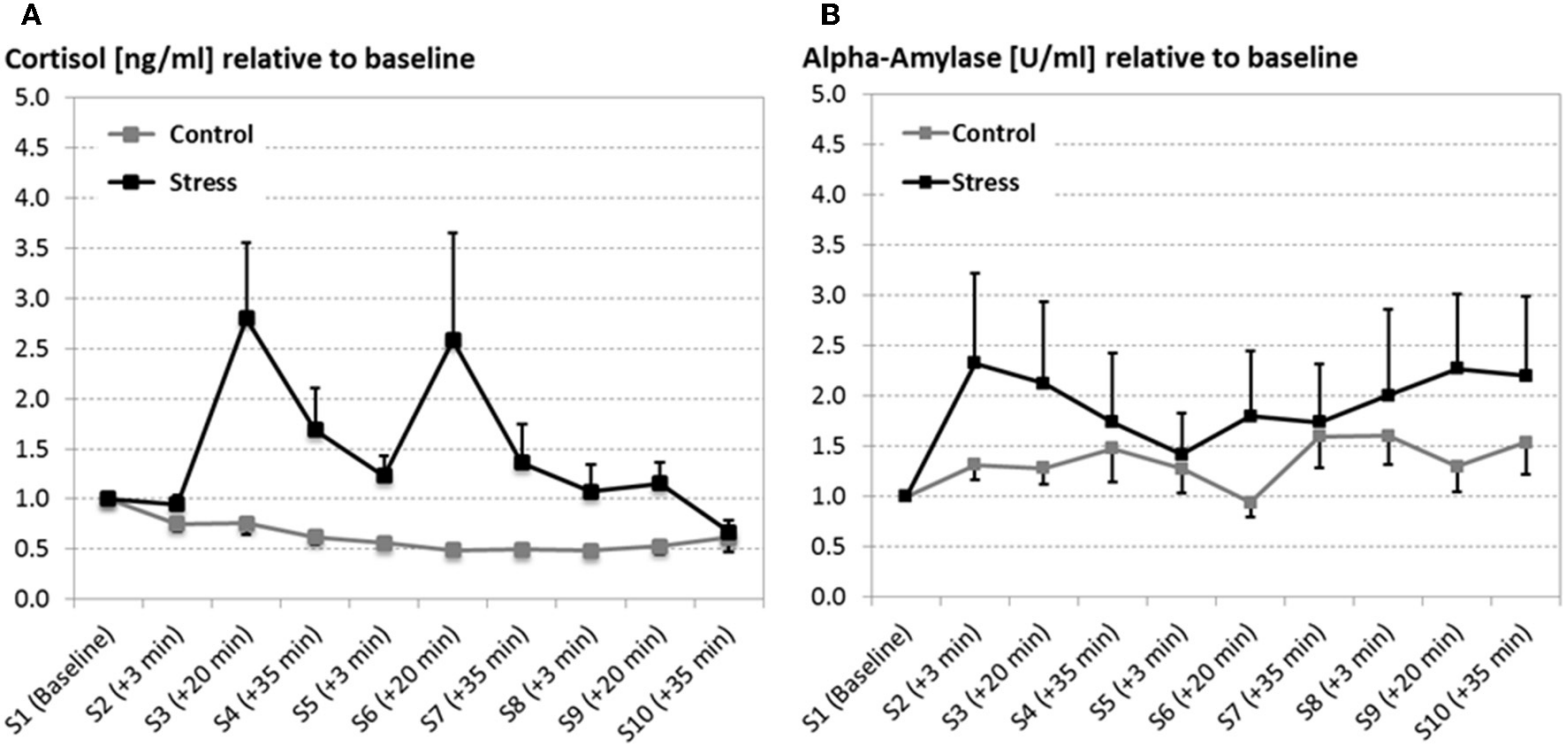
**Salivary cortisol in nanogram per milliliter (ng/ml; A) and alpha-amylase in units per milliliter (U/ml; B) at several time points (S1–S10) across the experiment (error bars depict the SE of the mean)**. Both, cortisol and alpha-amylase are reported as relative measures, which were qualified to the baseline measurement. After the baseline measurement the SECPT/warm water control procedure was conducted three times. For all experimental sessions 20 min after the SECPT procedure (S4, S7, and S9) cortisol and alpha-amylase responses were significantly increased in stressed participants compared to the warm water control group.

While both groups did not differ in the baseline cortisol measurement (*F* < 1) stress went along with an overall increase of cortisol compared to the control condition [*F*_(1, 22)_ = 5.451, *p* = 0.029, see Figure 2A]. Although the cortisol response seems to habituate over time, what is reflected by the main effect of the factor Experimental block [*F*_(2, 44)_ = 4.912, *p* = 0.024, ϵ = 0.693], the interaction Time by Group [*F*_(2, 44)_ = 4.183, *p* = 0.022] indicates that only stressed participants show an increase in cortisol levels for every experimental session (measurements 20 min after the SECPT/control, S3, S6, S9).

Although alpha-amylase data revealed a large variance, particularly for the stress group, the stress induction went along with a fast increase in alpha-amylase, which is quantified by the interaction of the factors Group by Time [*F*_(2, 44)_ = 4.538, *p* = 0.016; see Figure 2B].

### Behavioral data

For both groups error rates varied across the different Types of change, *F*_(3, 66)_ = 31.816, *p* < 0.001. While conditions that include a unilateral luminance change (LUM and LOU) did not show a significant difference (LUM: 16.71 %; LOU: 19.42 %; *p* = 0.915), most errors were committed for the conflict condition (30.83 %, all *p*'s < 0.001) and fewest for orientation changes (6.5 %, all *p*'s < 0.003).

**Figure 3:**
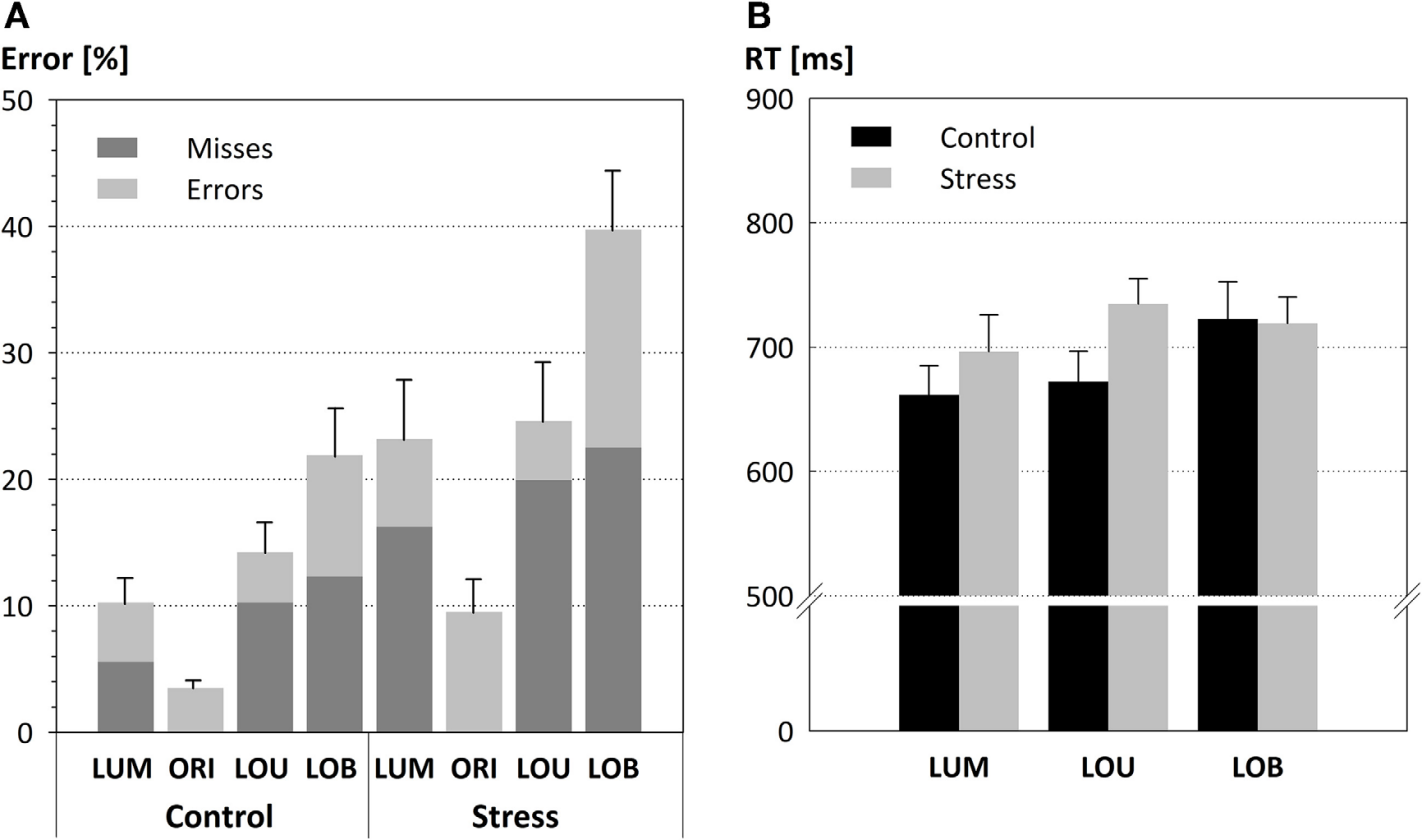
**Detection performance in form of mean error rates (A) and response times (B; error bars depict the SE of the mean)**. Both parameters varied with the strength of the conflict. Error rates but not response times are modulated by the SECPT manipulation. Thus, stress resulted in higher error rates, particularly in the conflict condition LOB. In addition, the stress induction led to an enhanced proportion of response misses (highlighted in dark gray).

Additionally, stress led to increased error rates (24.25 %) compared to the warm water control condition [12.48 %; *F*_(1, 22)_ = 7.609, *p* < 0.01, see Figure 3A]. *Post-hoc* comparisons show that this group difference was evident for the detection of unilateral luminance changes [LUM, *t*_(22)_ = 2,359, *p* < 0.028] and the conflict condition LOB [*t*_(22)_ = 2,838, *p* < 0.01], but not for orientation changes [ORI, *t*_(22)_ = 2,119, *p* = 0.055] and for trials with unilateral changes of both features [LOU, *t*_(22)_ = 1.796, *p* = 0.086].

Although there was no significant interaction between the Type of change and Group [*F*_(3, 66)_ = 1.946, *p* = 0.131] a significant trend in form of a cubic contrast indicates that the stress effect was most pronounced for the conflict condition (LOB) in which stressed participants committed 17.84% more errors than the warm water control group [*F*_(1, 22)_ = 4.37, *p* < 0.048].

It is also worth mentioning that response errors made by the stress group include a very large portion of misses (19.55 %) compared to the control group [9.38%; *F*_(1, 22)_ = 3.86, *p* = 0.062].

Response times (see Figure 3B) also revealed a main effect for the Type of change, *F*_(2, 44)_ = 4.062, *p* = 0.024, with longest response times for LOB (720 ms) compared to LOU (703 ms) and LUM (678 ms). The absence of a significant difference between the two groups, *F*_(1, 22)_ = 1.018, *p* = 0.324, and the missing interaction of the factors Type of change by Group, *F* < 1, indicated that the behavioral data pattern does not resemble a stress-induced performance-criterion shift from favoring accurate responses toward faster responses (i.e., a speed-accuracy trade-off).

### ERP data

**Figure 4:**
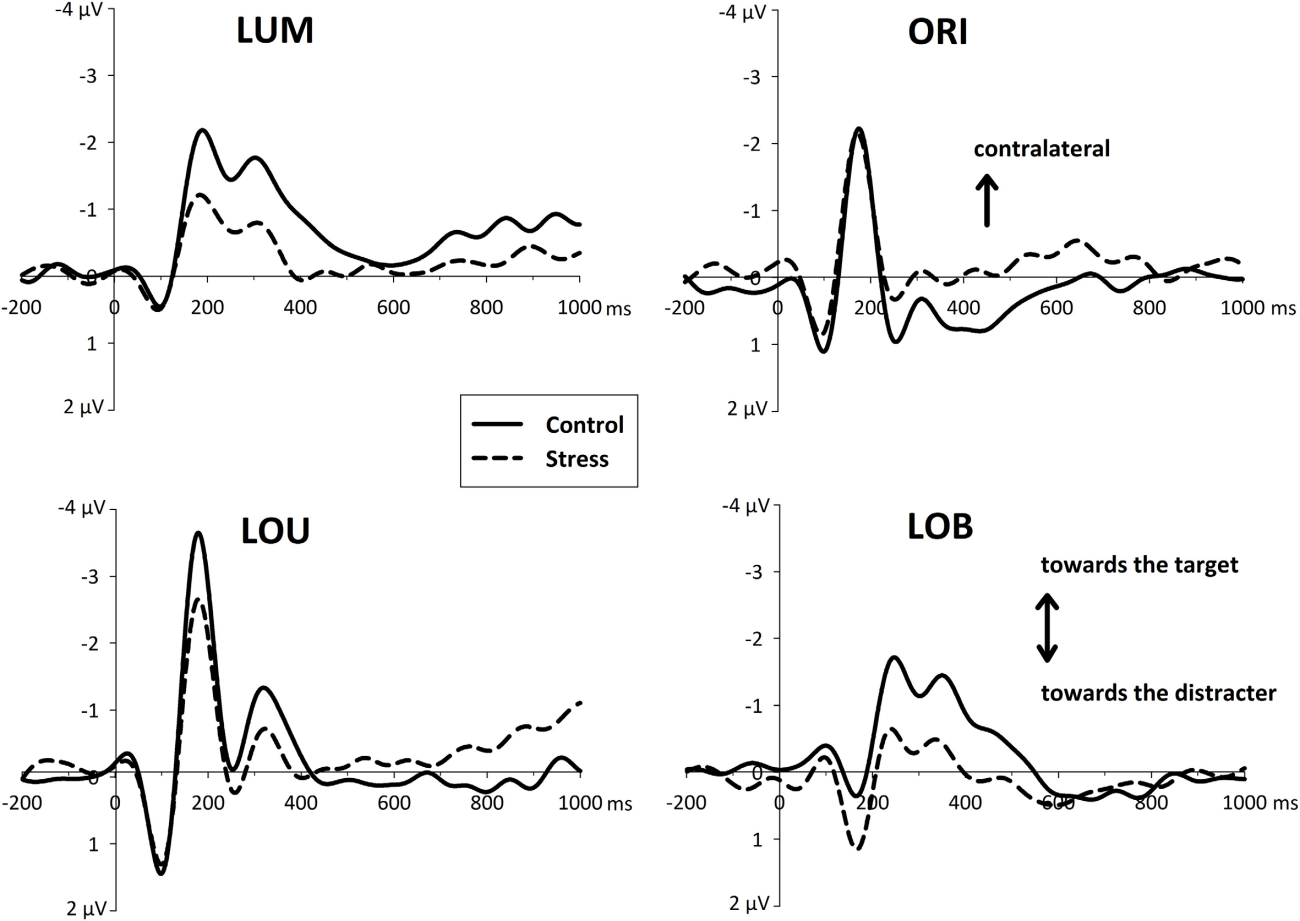
**Event-related asymmetries (ERL's, difference waves between activity contralateral and ipsilateral to an event) of the EEG over posterior electrodes (PO7/PO8) separately for each type of change (LUM, ORI, LOU, LOB)**. The increase of negativity contralateral to a unilateral transient (LUM, ORI, LOU) is plotted upward. For ERLs of the central conflict condition (LOB) negativity contralateral to the target stimulus is plotted upward. In the time window of the N1 ERLs indicate the capture of attention toward the more salient element. Subsequent asymmetries in the N2 range (N2pc) were decreased for the stressed participants.

ERLs revealed an asymmetric N1 that differed reliably from zero in all conditions [all *t*_(11)_ > 4.07, all *p*'s < 0.002, see Figure 4]. This posterior asymmetry in the N1 (N1pc) was also sensitive to the Type of change, with a contralateral negativity to the most salient stimulus of the display, *F*_(3, 72)_ = 98.84, *p* < 0.001, ϵ = 0.76. Whereas for the unilateral trials, LUM and LOU, the asymmetry in the N1 points to the relevant luminance change, for unilateral orientation changes (ORI) and for the bilateral conflict condition LOB, the N1 showed an enhanced activation contralateral to the irrelevant orientation change.

Additionally this early asymmetry, which is thought to reflect a first orientation of attention, was modulated by the stress manipulation, *F*_(1, 22)_ = 6.495, *p* < 0.02. Although there was no interaction of the Type of change by Group [*F*_(3, 66)_ = 1.214, *p* = 0.312], further pairwise comparisons showed that all unilateral conditions revealed a reduced N1pc for the stressed group [differences in amplitude for LUM: −0.85 μV and LOU: −1.16 μV; all *t*_(22)_ > 2.07, all *p*'s < 0.05] except orientation changes [differences in amplitude for ORI: −0.29 μV; *t*_(22)_ < 1, *p* = 0.45]. In contrast to that, in conflict trials stressed participant show an enhanced N1pc toward the irrelevant orientation change [differences in amplitude for LOB: +0.57 μV; *t*_(22)_ = 2.528, *p* = 0.019].

Around 300 ms a phasic N2 was visible contralateral to the position of the attended luminance change. While for the conditions with a spatially separated change in luminance (LUM and LOB) this N2pc differed reliably from zero [all *t*_(11)_ > 2.89, all *p*'s < 0.015], the other conditions LOU and ORI showed no reliable N2pc at all [all t_(11)_ < 1.98, all *p*'s > 0.073]. Furthermore, a significant group effect can only be shown for those conditions in which an N2pc was evident (LUM and LOB). This is supported by the interaction of Group by Type of change [*F*_(3, 66)_ = 3.822, *p* = 0.014]. *Post-hoc* pairwise comparisons show that the stress group show a reduced N2pc response for unilateral luminance changes [LUM: *t*_(22)_ = 2.61, *p* = 0.016] and for the conflict condition [LOB: *t*_(22)_ = 2.37, *p* < 0.02] but not for the remaining two conditions LOU and ORI [all *t*_(22)_ < 1.07, all *p*'s > 0.29].

**Figure 5:**
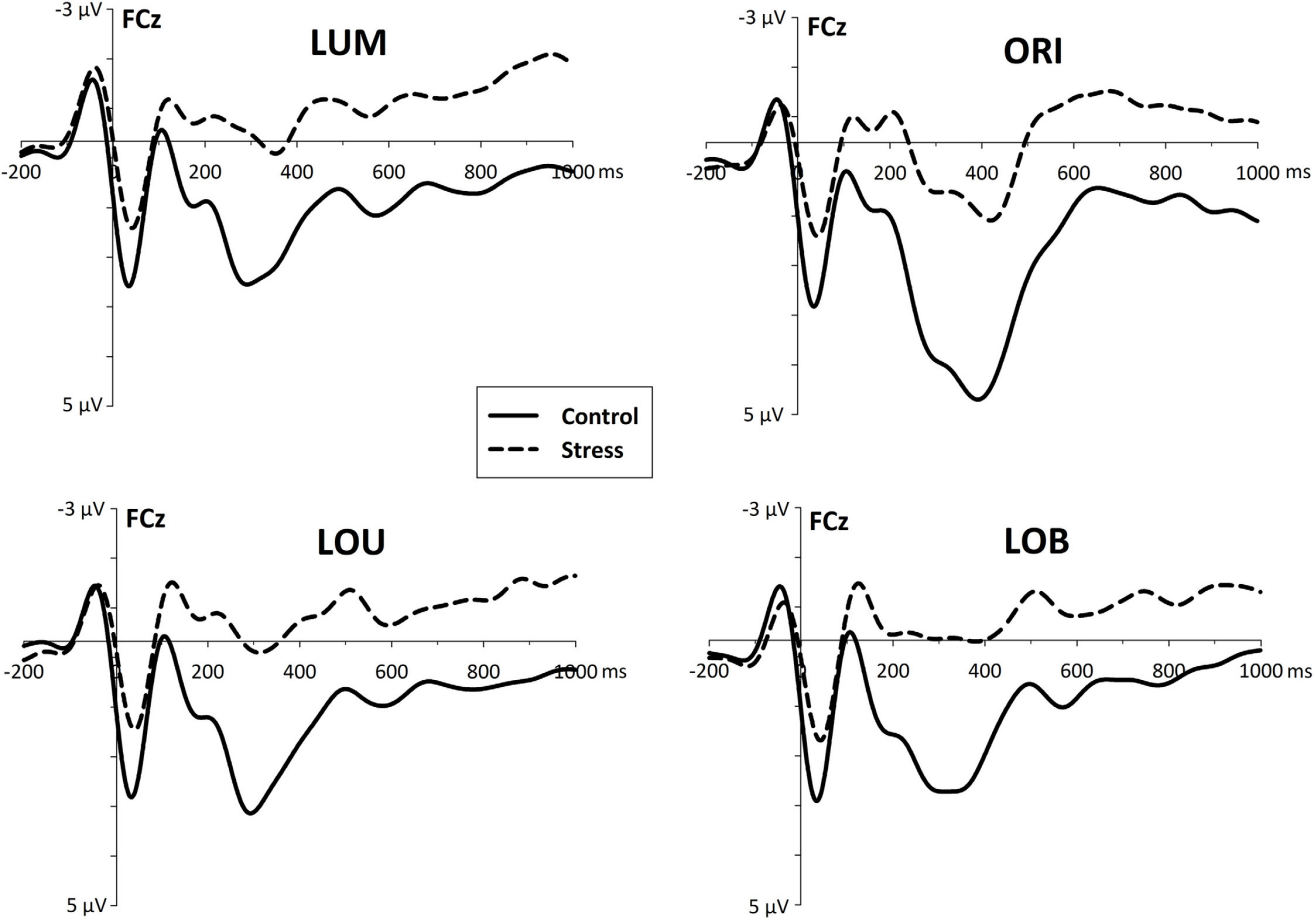
**Fronto-central event-related activity (ERP) derived from electrode site FCz, referred to linked mastoids**. For all conditions, stress went along with an increased fronto-central negativity which starts immediately after the presentation of the change (and possibly even before).

This differential posterior stress effects were accompanied by a fronto-central stress-related activity after the second stimulus display was presented. Whereas the control group revealed a differentiated activity with distinctive ERP components, the stress group shows an enhanced and rather sustained negativity in each condition (LUM, ORI, LOU, and LOB, see Figure 5).

In the early time window of the N2 (170–220 ms) stressed participants show an enhanced negativity [*F*_(1, 22)_ = 7.965, *p* < 0.01] compared to the control group. *Post-hoc* pairwise comparisons show, that this group difference was evident for all conditions (all *t*'s > 2.68, all *p*'s < 0.014).

While for stressed participants further EEG progression reveal no distinct ERP components that differ reliably from zero, 250–300 ms after the second stimulus display control subjects show a large P3a-like positivity [*F*_(1, 22)_ = 7.427, *p* < 0.02].

**Figure 6:**
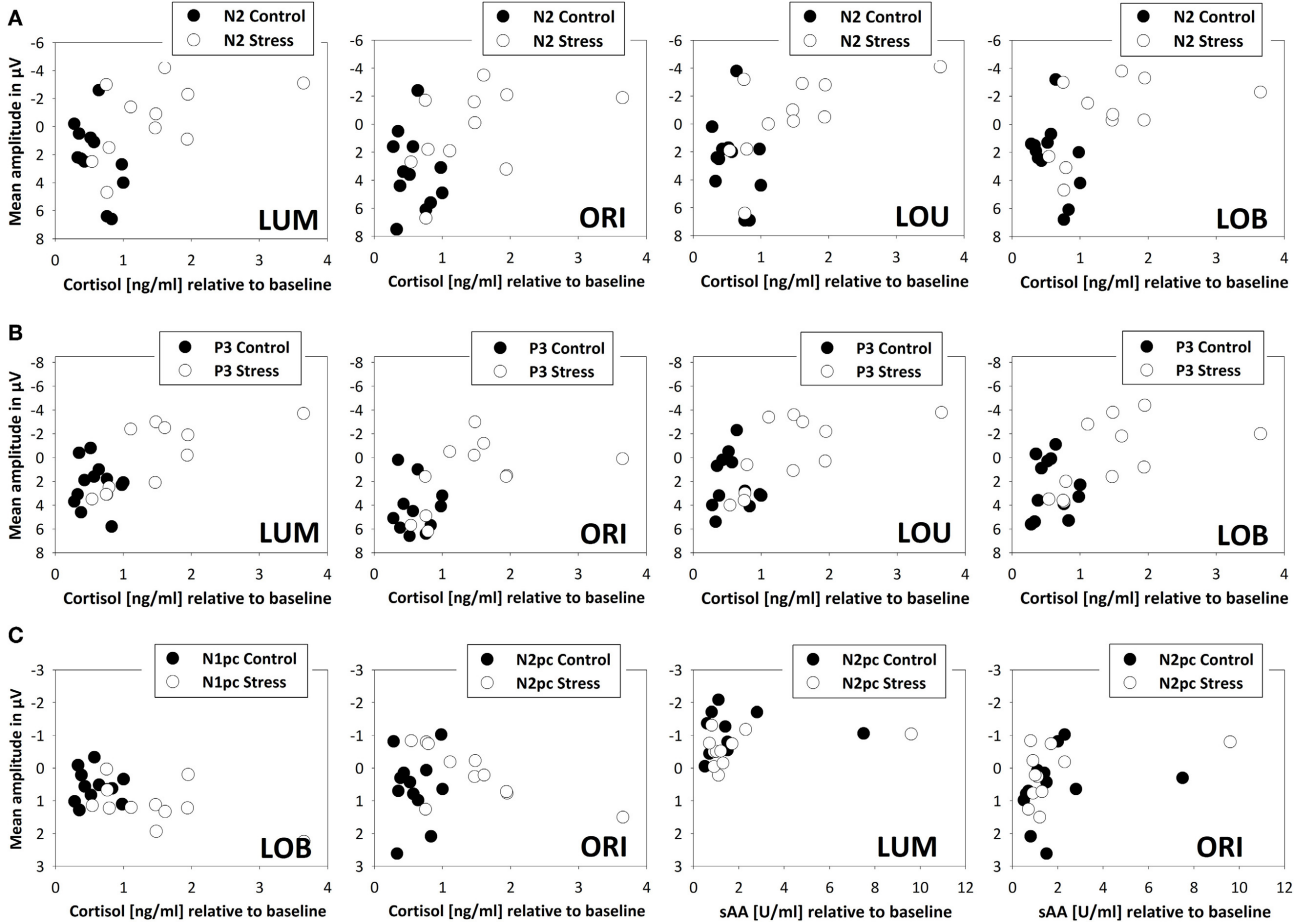
**Scatterplot denoting correlations between endocrine measures (salivary cortisol and alpha-amylase) and electrophysiological measures (ERPs and ERLs) for stressed (*N* = 11, open circles) and control participants (*N* = 12, filled circles)**. All scatterplots exclude one participant of the stress group, because of its high absolute and relative cortisol levels. Fronto-central brain activity 20 min after the SECPT/warm water control (+20 min) is indicated by the N2 **(A)** and P3 **(B)**. Posterior attentional processing, indicated by the N1pc and N2pc is shown in row **(C)**. Reliable correlations between sAA and N2pc were evident directly after the SECPT/warm water control (+3 min) only.

Across all subjects, correlations with the fronto-central brain activity underline the negative effects of stress on cognitive control functioning. At all measurement time points (+3 min, +20 min, and +35 min) increases in salivary cortisol went along with an increased fronto-central N2 for all Types of change (all ρ's > −0.370, *N* = 23, all *p*'s < 0.041, see Figure 6).

Restricting the analysis to stressed participants, this correlation got even stronger (all ρ's > −0.545, *N* = 11, *p* < 0.041). As obvious from Figure 6, this relation disappeared when control participants were tested alone (all ρ's < 0.399, *N* = 12, all *p*'s > 0.10). For all Types of change an additional correlation between stress-induced salivary cortisol levels and the P3 amplitude at all measurement time points was evident (all ρ's > −0.541, N = 23, all *p*'s < 0.004). The higher the cortisol levels, the smaller the P3 amplitude. When calculating correlations for the two experimental groups separately, a significant interrelation was only evident for stressed participants (stress: all ρ's > −0.755, *N* = 11, all *p*'s < 0.004; warm water control: all ρ's < −0.224, *N* = 12, *p* > 0.242).

Twenty min after the stressor a relation between cortisol levels and the initial allocation of attention, by means of the N1pc, was evident for the conflict condition only (ρ = 0.418, *N* = 23, *p* < 0.024). The following N2pc, which can be seen as an index for the expression of top-down control, was significantly correlated to salivary cortisol levels in trials with orientation changes. This interrelation was evident +3, +20, and +35 min after the stress induction (all ρ's > −0.627, *N* = 11, all *p*'s < 0.019) but not in the warm water control group (all ρ's < −0.175, *N* = 12, all *p's* > 0.293).

Although levels of salivary alpha-amylase were not related to the behavioral performance or fronto-central brain activity, immediately after the stress induction (+3 min) it correlated with the N2pc amplitude in trials where a unilateral luminance or orientation change had to be detected (LUM: ρ = −0.357, *N* = 23, *p* < 0.047; ORI: ρ = −0.447, *N* = 23, *p* < 0.016).

## Discussion

The purpose of the present study was to examine the influence of stress on attentional selection, particularly when the spatial conflict is high. Therefore, half of the male sample was exposed to acute stress by the SECPT procedure (Schwabe et al., 2008) before performing a change detection-like task. In this task they had to respond to luminance changes of the stimuli and to ignore very salient orientation changes.

As intended, only the stress group displayed an enhanced activity of the SNS immediately after the first stress exposure, indicated by an increase in salivary alpha-amylase, which continued to increase over the experiment. Furthermore, the stress group showed elevated cortisol levels from 20 min after each stress cessation compared to the warm water control group. However, although the cortisol release decreased from the first to the third session, this is in line with the notion that the repeated exposure with a stressor leads to a habituation/decrement of the HPA activity, which end up in decreasing cortisol responses (Kovács et al., 2005). Thus, given the potential limitation of the chosen sample that include only men, these results indicate the typical immediate SNS-related and prolonged HPA-mediated stress responses indicating a successful stress induction.

Moreover, the stress group showed increased error rates compared to the control group, particularly for the conflict condition and for luminance changes. These are conditions in which the less salient luminance change is presented solely or in competition to a more salient orientation change. The increased error rates and response times in stressed individuals compared to controls are unlikely to be due to a speed-accuracy trade-off. If stress resulted in premature responding, stressed individuals compared to controls should display smaller mean RTs on the one hand and increased error rates not only for conflict trials but also for non-conflict trials on the other hand. Since the behavioral data pattern did not resemble one of those assumptions, such a stress-induced strategic performance-criterion shift from favoring accurate responses toward faster responses (i.e., a speed-accuracy trade-off) seems to be unlikely. On the contrary, the findings rather point toward a specific effect of acute stress on task performance. Stressed individuals seemed to display a mis-weighting of the relevant feature that had to be detected. The fact that increased error rates for the stress group included a very large portion of response misses even emphasize a lack of top-down controlled selection bias toward the less salient target feature (the luminance of a bar).

This result fits with the fundamental principles of competitive interactions that the enhancement of one stimulus occurs at the expense of other stimuli in the display (Somers et al., 1999; Pinsk et al., 2004; Gazzaley et al., 2005). Furthermore, it nicely shows that under conditions of stress attentional selection changes from top-down controlled to more bottom-up driven selection (Arnsten, 2009; e.g., Liston et al., 2009).

The behavioral data pattern was supported by the electrophysiological data. While for the condition, which did not include a target object—orientation changes—the posterior and asymmetric N1 did not show a stress-related difference, the stress group showed a decreased N1pc for the relevant luminance change. Furthermore, in the conflict condition (LOB) the N1pc was even enhanced but contralateral to irrelevant orientation change. Thus, the data pattern suggests that the initial attentional orientation toward the relevant feature, the luminance, was weakened by the stressor. Moreover, the data suppose that in case of a spatial attentional conflict the stress group even amplified and concentrated on the irrelevant feature, the orientation changes.

The results of Shackman et al. (2011) show an overall stress-induced amplification of the visual N1 which is suggested to be due to an increased gain or sensory processing. Furthermore, in an MEG study by Elling et al. (2012) anticipatory stress caused an enhanced distractibility in the visual modality and was accompanied by an N1m source strength increase under stressor anticipation in a male sample. However, in the study of Shackman et al. (2011) the electrophysiological finding of the N1 unfortunately neither disentangle relevant from irrelevant stimulus processing within the framework of attentional competition or focused on the post-selective processes, nor did they report a continuous measure of the stress response or show an equivalent in the behavioral performance.

Regarding the fact that attention had not been initially allocated to the relevant change, based on activation in the N1 time window, a subsequent top-down bias in favor of the target should be required for successful target change detection (Schankin and Wascher, 2007; Schneider et al., 2012a,b). However, this should not be the case if participants are stressed.

Contrary to our expectations, this component, which is thought to reflect a reorienting to the relevant feature, was evident in stressed participants as well. However, this was only true in conditions in which the initial attentional selection of the less salient stimulus feature might not have been successful, i.e., LUM and LOB. Furthermore, this N2pc was decreased for the stressed participants. Thus, stressed participants show an early mis-weighting of the relevant luminance changes, indicated by the differential deflection of the N1pc. Additionally, the stress-induced alteration of the subsequent N2pc reflects a strong attenuation of top-down control and post-selective target filtering.

An increased early fronto-central activity for the stress group in the time window of the N2 indicates an increased effort to control the selection and to solve the task successfully. However, when participants are stressed this increased effort did not have sufficient impact on attentional selection, what might be caused by an impaired transmission from frontal brain structures to the relevant posterior areas and resulted in a reduced N2pc. This result is in line with findings of other studies (e.g., Liston et al., 2009; Qin et al., 2009) which could show that the functional connectivity between the PFC and other brain regions was reduced under stress.

Further EEG progression over fronto-central areas reveal a lack of a differential task processing when participants got stressed. The P3a generation, which serves as an indicator of the integrity of frontal lobe functioning (Knight, 1984; Knight et al., 1995) was almost absent in stressed participants. This is in line with findings of Shackman et al. (2011), who found a stress-related attenuation of the P3 in a speeded visual discrimination task.

P3 is modulated by different arousal levels (Pribram and McGuiness, 1975). Thereby, P3a can be related to frontal focal attention and is mediated by dopaminergic activity, whereas P3b is related to attention resource allocation (Intriligator and Polich, 1995; Kok, 1997) in temporal-parietal areas and mediated by noradrenaline release of the LC (Pineda et al., 1989; Braver and Cohen, 2000; Nieuwenhuis et al., 2005). Thus, as stress affected frontal lobe functioning as well as activity of temporal-parietal areas via the LC, both subcomponents (P3a and P3b) are assumed to be altered in situations of stress.

While in the present study a direct relation between stress and behavioral performance was absent and correlations with markers of the posterior attention network (N1pc and N2pc) seem to be rather complex, stress can be directly linked to frontal lobe functioning. By that increased cortisol levels went along with an overall enhanced fronto-central N2 as well as a reduction/lack of a P3a.

Animal studies show dissociations between psychosocial (e.g., in a social defeat situation) and physiological (e.g., due to foot shocks) effects of stress (Gesing et al., 2001; Kavushansky et al., 2009). And also field studies suggest that additional psychological factors influence attention performance in the context of stress (e.g., Sliwinski et al., 2006). By that stress associated intrusive thoughts negatively impact on attentional resources, particularly in demanding tasks. However, with our design the question as to whether or not the impairment in cognitive control functioning is finally attributable to the endocrine, the affective changes, or an interaction of both remains unanswered. It is likely that those factors interact at multiple levels (e.g., Abercrombie et al., 2006; Schwabe et al., 2008).

In conclusion, indicated by the N1pc, situations of acute stress led to a mis-weighting of the relevant stimulus feature so that irrelevant but very salient orientation changes dominated the initial processing stage. A subsequent top-down bias toward the luminance target in the form of an N2pc was required for successful change detection. However, this top-down controlled re-allocation of attentional resources was decreased or even absent in conditions of stress. The present study additionally shows a direct interrelation between stress-related cortisol levels and PFC functioning. Although enhanced early fronto-central brain activity suggests that stressed participants show an increased effort to solve the task, this did not have any impact on the task performance and further progression of the ERP did not reveal any differentiated task processing. Thus, stressed participants were not able to maintain the intentional control to bias the competition toward the less salient but relevant target feature, which resulted in increased response errors, particularly when the spatial attentional conflict was high.

### Conflict of interest statement

The authors declare that the research was conducted in the absence of any commercial or financial relationships that could be construed as a potential conflict of interest.
